# Supplementary material for: Causal interplay between lactose intolerance and gut microbiota: a combined bidirectional Mendelian randomization and in vivo validation study
Source: Front Nutr. 2026 Jun 1;13:1803337. doi: 10.3389/fnut.2026.1803337 (PMC13265576; doi:10.3389/fnut.2026.1803337)
Supplement: Supplementary file 1 [file Data_Sheet_1.zip › supplementary materials/Forward/leave one out cross validation/ebi-a-GCST90027798.finngen_R12_E4_LACTONAS.pdf]

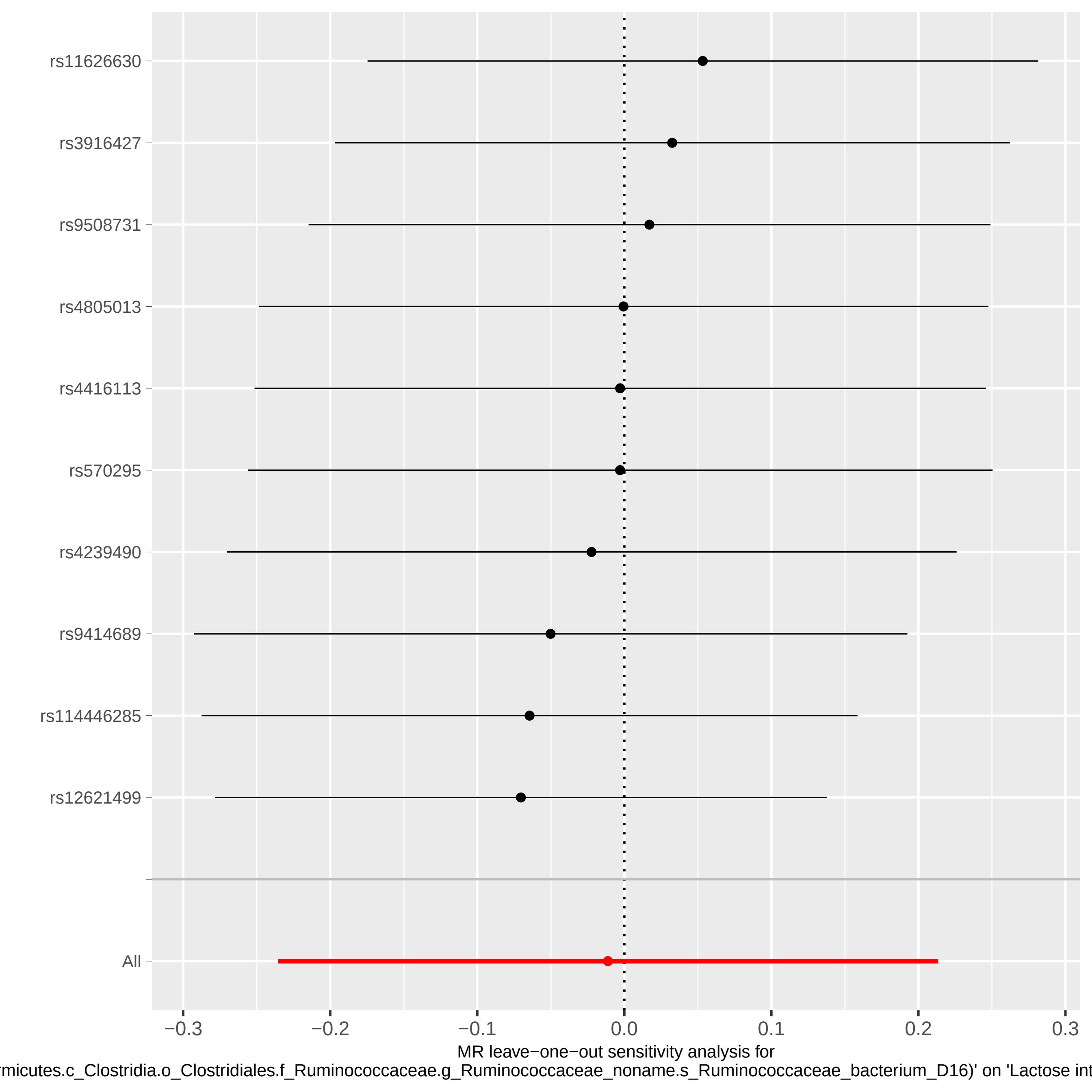

rs11626630
rs3916427
rs9508731
rs4805013
rs4416113
rs570295
rs4239490
rs9414689
rs114446285
rs12621499
All
−0.3
−0.2
−0.1
0.0
0.1
0.2
0.3
MR leave−one−out sensitivity analysis for
rmicutes.c_Clostridia.o_Clostridiales.f_Ruminococcaceae.g_Ruminococcaceae_noname.s_Ruminococcaceae_bacterium_D16)' on 'Lactose int
